# A proteomic repertoire of autoantigens identified from the classic autoantibody clinical test substrate HEp-2 cells

**DOI:** 10.1186/s12014-020-09298-3

**Published:** 2020-09-21

**Authors:** Julia Y. Wang, Wei Zhang, Jung-hyun Rho, Michael W. Roehrl, Michael H. Roehrl

**Affiliations:** 1 Curandis, New York, USA; 2 Department of Gastroenterology, Affiliated Hospital of Guizhou Medical University, Guizhou, China; 3 MP Biomedicals New Zealand Limited, Auckland, New Zealand; 4 Department of Pathology, Memorial Sloan Kettering Cancer Center, New York, USA; 5 Human Oncology and Pathogenesis Program, Memorial Sloan Kettering Cancer Center, New York, USA

**Keywords:** Autoantigen, Autoantibody, HEp-2 cell, Dermatan sulfate, Antinuclear autoantibody, ANA test, Autoantibody test

## Abstract

**Background:**

Autoantibodies are a hallmark of autoimmune diseases. Autoantibody screening by indirect immunofluorescence staining of HEp-2 cells with patient sera is a current standard in clinical practice. Differential diagnosis of autoimmune disorders is based on commonly recognizable nuclear and cytoplasmic staining patterns. In this study, we attempted to identify as many autoantigens as possible from HEp-2 cells using a unique proteomic DS-affinity enrichment strategy.

**Methods:**

HEp-2 cells were cultured and lysed. Total proteins were extracted from cell lysate and fractionated with DS-Sepharose resins. Proteins were eluted with salt gradients, and fractions with low to high affinity were collected and sequenced by mass spectrometry. Literature text mining was conducted to verify the autoantigenicity of each protein. Protein interaction network and pathway analyses were performed on all identified proteins.

**Results:**

This study identified 107 proteins from fractions with low to high DS-affinity. Of these, 78 are verified autoantigens with previous reports as targets of autoantibodies, whereas 29 might be potential autoantigens yet to be verified. Among the 107 proteins, 82 can be located to nucleus and 15 to the mitotic cell cycle, which may correspond to the dominance of nuclear and mitotic staining patterns in HEp-2 test. There are 55 vesicle-associated proteins and 12 ribonucleoprotein granule proteins, which may contribute to the diverse speckled patterns in HEp-2 stains. There are also 32 proteins related to the cytoskeleton. Protein network analysis indicates that these proteins have significantly more interactions among themselves than would be expected of a random set, with the top 3 networks being mRNA metabolic process regulation, apoptosis, and DNA conformation change.

**Conclusions:**

This study provides a proteomic repertoire of confirmed and potential autoantigens for future studies, and the findings are consistent with a mechanism for autoantigenicity: how self-molecules may form molecular complexes with DS to elicit autoimmunity. Our data contribute to the molecular etiology of autoimmunity and may deepen our understanding of autoimmune diseases.

## Background

Autoimmune diseases occur when an immune system starts to attack its own body. Any tissue may become a target of autoimmune attacks, which is why autoimmune diseases constitute a wide spectrum of symptoms, with more than 100 autoimmune diseases having been classified thus far. The underlying mechanism of autoimmunity has been rather intriguing. Normally, the immune system reserves immune responses to attack invading microorganisms and protect the body. Due to unclear circumstances, however, the immune response can sometimes go astray and mistakenly attack its own tissue. From a molecular point of view, certain self-molecules are targeted, as if they are non-self, by autoreactive cells, autoantibodies (autoAbs), or other factors, which subsequently leads to damage of the tissue where the autoantigens (autoAgs) reside.

Among the hundreds of thousands of human molecules, only a small portion have been reported to be autoAgs. Moreover, the autoAgs appear to be a random collection of molecules that are expressed in different parts of human body and exhibit various biological functions. Thus, it is mysterious how these different molecules can all trigger a similar cascade of autoimmune responses. A key, missing mechanism is how non-antigenic self-molecules become autoantigenic non-self. Theoretically, a molecule may change itself in many ways by alternating its chemical composition or biochemical properties. A protein may change by mutation, glycosylation, phosphorylation, methylation, citrullination, or fusion with another protein. While simple compositional or structural changes can certainly generate a huge random mix of altered molecules, it cannot explain how they may induce autoimmune responses, let alone similar ones.

Based on our studies, we have proposed a uniform autoantigenicity mechanism by which autoAgs share a common biochemical property in their affinity to dermatan sulfate (DS), and by which autoAgs forming a molecular complex with DS to induce autoreactive B cell responses [1–5]. DS, a glycosaminoglycan polysaccharide, is expressed most abundantly in the skin and other connective tissues [6], and its expression has been reported to increase during high cellular turnaround, such as wound healing [7–9]. It is possible that DS is upregulated to facilitate dead cell clearance and new cell growth for tissue regeneration. We found that, when cells are dying or under stress, they express certain self-molecules to which DS has peculiar affinity [1]. By forming complexes with DS, these self-molecules are transformed from non-antigenic singular molecules to antigenic complexes. Furthermore, these DS-autoAg complexes are capable of stimulating autoreactive CD5 B cell proliferation and differentiation [1], likely via PKC- and PI3K-dependent signaling pathways [10, 11].

To relate our findings to clinical utilities, this study examined proteins from HEp-2 cells, the classic substrate in the routine clinical tests for antinuclear autoantibodies (ANAs) [12]. HEp-2 cells were chosen for clinical tests because of their human origin, high mitotic activity, and ability to induce expression of clinically important autoantigens such as mitotic nuclear autoantigens. AutoAbs in patient sera are typically detected by indirect fluorescence staining of HEp-2 cells, and clinical diagnoses are based on about 30 commonly recognizable staining patterns, e.g., nuclear, cytoplasmic, homogenous, or speckled [12]. Despite its clinical popularity, this test lacks molecular details of the autoAg targets, as it is not known exactly which autoAgs are recognized by the autoAbs or whether a single or several autoAgs are recognized. Further tests of specific autoAgs by molecular assays such as ELISA are often performed using proteins extracted from HEp-2 cells or other means. This study was thus pursued to identify the repertoire of autoAgs produced by HEp-2 cells for development of future molecular clinical tests.

## Methods

### HEp-2 cell culture and protein extraction

HEp-2 cell line was obtained from ATCC (Manassas, VA, USA). HEp-2 cells were cultured in Eagle’s Minimum Essential Medium supplemented with 10% fetal bovine serum (ThermoFisher Scientific) and penicillin–streptomycin-glutamine mixture (ThermoFisher Scientific) at 37 °C in 75 cm^2^ tissue culture flasks. About 100 million cells were harvested and used for protein extraction. Harvested cells were suspended in 10 mL of 50 mM phosphate buffer (pH 7.4) containing the Roche Complete Mini protease inhibitor cocktail (Sigma Aldrich). Cells were homogenized on ice with a microprobe sonicator until the turbid mixture became nearly clear with no visible cells left. The homogenate was centrifuged at 10,000*g* at 4 °C for 20 min, and the supernatant was collected as the total protein extract. Protein concentrations were measured with the Bio-Rad RC DC protein assay.

### DS-Sepharose resin preparation

Dermatan sulfate (DS) (Sigma-Aldrich) were covalently coupled to EAH Sepharose 4B resins (GE Healthcare) as previously described [2–4]. Twenty-mL Sepharose 4B resins were washed with distilled water and then with 0.5 M NaCl solution. The resins were mixed with 100 mg of DS that was pre-dissolved in 10 mL of 0.1 M MES buffer (pH 5.0). The mixture was then added 0.58 g of N-(3-dimethylaminopropyl)-N’-ethylcarbodiimide hydrochloride (Sigma-Aldrich). The coupling reaction was carried out by end-over-end tube rotation at 25 °C for 24 h. After the first 60 min, the pH of the reaction mixture was adjusted to 5.0 with 0.1 M NaOH. After the coupling, the resins were washed three times, first with a low-pH buffer (0.1 M acetate, 0.5 M NaCl, pH 5.0) and then with a high-pH buffer (0.1 M Tris, 0.5 M NaCl, pH 8.0). The DS-Sepharose resins were packed in 10 mM phosphate buffer (pH 7.4) into a C 16/20 FPLC column (GE Healthcare Life Sciences).

### DS-affinity fractionation

The total proteins extracted from HEp-2 cells were fractionated in a DS-Sepharose column with a BioLogic Duo-Flow system (Bio-Rad). About 40 mg of proteins in 40 mL of 10 mM phosphate buffer (pH 7.4; buffer A) were loaded into the DS-Sepharose column at a rate of 1 mL/min. After loading, the column was washed with 60 mL of buffer A to remove excess and non-binding proteins, followed by eluting with 40 mL of 0.2 M NaCl in buffer A to further remove very weakly binding proteins. Proteins with low to high DS-affinity were eluted with sequential 40-mL step gradients of 0.4 M, 0.6 M, and 1.0 M NaCl in buffer A. Fractions were desalted and concentrated to 0.5 mL with 5-kDa cut-off Vivaspin 20 centrifugal filters (Sartorius). The protein concentration of each fraction was measured. Fractionated proteins were further separated by 1-D SDS PAGE using 4–12% NuPAGE Novex Bis–Tris gels with MES running buffer (Invitrogen). Each gel lane was divided into three sections and subjected to protein sequencing.

### Mass spectrometry sequencing

Protein sequencing was performed at the Taplin Biological Mass Spectrometry Facility at Harvard Medical School (Boston, MA, USA). Protein gel sections were cut into 1-mm^3^ pieces, dehydrated with acetonitrile, and dried in a speed-vac. The gel pieces were rehydrated with 50 mM NH_4_HCO_3_ containing 12.5 μg/mL modified sequencing-grade trypsin (Promega) at 4 °C for 45 min. Tryptic peptides were separated on a nano-scale C_18_ HPLC capillary column and analyzed after electrospray ionization in an LTQ linear ion-trap mass spectrometer (Thermo Scientific). Peptide sequences and protein identities were assigned by matching the measured fragmentation patterns with protein or translated nucleotide databases using Sequest software. Peptides were required to be fully tryptic peptides with XCorr values of at least 1.5 for 1^+^ ion, 1.5 for 2^+^ ion, or 3.0 for 3^+^ ion. All data were manually inspected. Only proteins with ≥ 2 peptide matches were considered positively identified.

### Literature search for autoantigen confirmation

Extensive literature searches with PubMed were carried out to identify whether proteins identified in this study had been previously reported as autoantibody-targeted autoantigens. Keywords in the searches included the protein name and the MeSH term “autoantibodies.” When a particular protein name did not yield any results, alternative protein names were obtained from the Uniprot database and used as keywords for repeated searches. In the case of no results, gene names and alternative gene names were used as additional keywords in searches. When multiple reports for an autoantigen were found, reports with the most relevance or open access and free text were preferably cited in this paper.

### Pathway and process enrichment analysis by Metascape

The collection of DS-affinity enriched proteins identified from this study was analyzed with Metascape, a gene annotation and analysis resource [13]. Pathway and process enrichment analysis was carried out with KEGG Pathway, GO Biological Processes, Reactome Gene Sets, Canonical Pathways, CORUM, TRRUST, DisGeNET, and PaGenBase. All genes in the genome were used as the enrichment background. Terms with a *p* value < 0.01, a minimum count of 3, and an enrichment factor (ratio between the observed counts and the counts expected by chance) > 1.5 were collected and grouped into clusters based on their membership similarities. p-values were calculated based on the accumulative hypergeometric distribution, and q-values were calculated using the Banjamini-Hochberg procedure to account for multiple testings. Kappa scores were used as the similarity metric when performing hierarchical clustering on the enriched terms, and sub-trees with a similarity of > 0.3 were considered a cluster. The most statistically significant term within a cluster was chosen to represent the cluster. Protein–protein interaction enrichment analysis was carried out with BioGrid, InWeb_IM, and OmniPath. The resultant network contained the subset of proteins that form physical interactions with at least one other member in the list. The Molecular Complex Detection (MCODE) algorithm was applied to identify densely connected network components. Pathway and process enrichment analysis was applied to each MCODE component independently, and the three best-scoring terms by p-value have been retained as the functional description of the corresponding components.

### Protein–protein association network analysis by STRING

The collection of proteins identified from this study were also analyzed with STRING, a database of known and predicted protein–protein interactions [14]. Specific and meaningful protein–protein associations represent proteins jointly contributing to a shared function, but not necessarily physically binding to each other. The database currently covers 24,584,628 proteins from 5090 organisms. Known interactions are sourced from curated databases (metabolic pathways, protein complexes, signal transduction pathways, etc.), experimental evidence (co-purification, co-crystallization, Yeast2Hybrid, genetic interactions, etc.), gene neighborhoods (groups of genes that are frequently observed in each other’s genomic neighborhood), gene fusions (genes that are sometimes fused into single open reading frames), gene co-occurrence (gene families whose occurrence patterns across genomes show similarities), gene text mining (automated, unsupervised searching for proteins that are frequently mentioned together), and co-expression (proteins whose genes are observed to be correlated in expression, across a large number of experiments).

## Results

### DS-affinity fractionation enriches certain HEp-2 proteins

Proteins were extracted from freshly cultured HEp-2 cells and fractionated with DS-affinity resins. Since DS molecules are poly-anionic, ionic interactions are expected to be a main contributor to DS affinity. We therefore developed an NaCl salt step-gradient method to sequentially dissociate and elute proteins with from DS resins. The identities of proteins in each DS-affinity enriched fraction were obtained by mass spectrometry sequencing.

### Proteins with high DS-affinity

From fractions eluted with 1.0 M NaCl from DS-affinity resins, only 7 proteins were identified by mass spectrometry sequencing (Table 1). These include two histone proteins (H4 and H2BE), Scl-70, and Ro/SS-A, all of which are among the most classical nuclear autoantigens (Table 1). Three isoforms of ribosomal proteins are also identified, including 60S ribosomal proteins (L6 and L7) and 40S ribosomal protein S9. Ribosomal proteins are also a class of well-known autoantigens, and heterogenous forms have been reported to be recognize autoantibodies, however, it is not clear exactly which and how many isoforms are autoantigens. L6 and L7 have been reported as autoantigens, whereas S9 awaits further confirmation (Table 1).

**Table 1:** Proteins identified from DS-affinity enrichment of HEp-2 cell extract

Pept. #^a^	Gene	Protein	H	M	L	pI	Ref.
		Nuclear/Chromosome/Mitotic Cell Cycle					
10	HIST1H4A	Histone H4	+	+		11.36	[18]
3	HIST2H2BE	Histone H2B type 2-E	+	+		10.31	[19]
4	H1-2	Histone H1.2 (H1F2, HIST1H1C)		+		10.94	[20]
3	H2AFY	Core histone macro-H2A.1		+		9.80	[21]
2	H1-5	Histone H1.5 (H1F5, HIST1H1B, DNMT3A)		+		10.91	[20]
2	H1F0	Histone H1.0		+		10.84	[22]
2	H2AFV	Histone H2A.V		+		10.58	[23]
2	HIST2H3A	Histone H3.2		+		11.27	[24]
Ab	TOP1	DNA topoisomerase I (Scl-70 autoantigen)	+				[2, 25]
5	SET	Protein SET (Inhibitor of granzyme A-activated DNase, HLA-DR-associated protein II)		+		4.22	?
Ab	TRIM21	E3 ubiquitin-protein ligase TRIM21 (Ro/SS-A autoantigen)	+				[2, 26]
4	SSB	Lupus La protein		+	+	6.68	[27]
4	ANP32B	Acidic leucine-rich nuclear phosphoprotein 32 family member B (putative HLA-DR-associated protein I-2)		+		3.93	?
2	PRMT1	Protein arginine N-methyltransferase 1 (Histone-arginine N-methyltransferase PRMT1)			+	5.18	?
28	DDB1	Damage-specific DNA-binding protein 1			+	5.14	[2]
2	NPM1	Nucleophosmin		+		4.64	[28]
4	KPNB1	Importin subunit beta-1 (pore targeting complex 97 kDa subunit)			+	4.68	[29]
3	PTMA	Prothymosin alpha			+	3.66	[30]
3	HDGF	Hepatoma-derived growth factor (High mobility group protein 1-like 2)			+	4.70	[31]
12	ENO1	Alpha-enolase			+	7.01	[2, 32]
3	MYBBP1A	Myb-binding protein 1A		+		9.34	?
4	YBX3	Y-box-binding protein 3 (Single-strand DNA-binding protein)		+		9.77	[2, 33]
10	YWHAE	14-3-3 protein epsilon			+	4.63	[34]
5	YWHAZ	14-3-3 protein zeta/delta			+	4.73	[35]
4	YWHAG	14-3-3 protein gamma			+	4.80	[34]
3	YWHAQ	14-3-3 protein theta			+	4.68	[36]
2	YWHAB	14-3-3 protein beta/alpha (Protein kinase C inhibitor protein 1)			+	4.76	?
4	SFN	14-3-3 protein sigma (Stratifin)			+	4.68	?
Ab	PCNA	Proliferating cell nuclear antigen		+			[2, 37]
21	XRCC6	Ku70 (X-ray repair cross-complementing protein 6, ATP-dependent DNA helicase 2 subunit 1)		+	+	6.23	[38]
10	XRCC5	Ku80 (Ku86, X-ray repair cross-complementing protein 5, ATP-dependent DNA helicase 2 subunit 2)		+	+	5.55	[39]
4	PRKDC	DNA-dependent protein kinase catalytic subunit (DNA-PKcs)		+		6.75	[40]
2	MCM6	DNA replication licensing factor MCM6			+	5.28	?
2	MCM2	DNA replication licensing factor MCM2			+	5.34	?
2	MCM3	DNA replication licensing factor MCM3			+	5.53	?
		RNA Metabolism/Ribonucleoprotein Granule					
3	RPL6	60S ribosomal protein L6	+			10.59	[41]
2	RPS9	40S ribosomal protein S9	+			10.66	[42]
2	RPL7	60S ribosomal protein L7	+			10.66	[43]
16	RPL5	60S ribosomal protein L5		+		9.73	[44]
2	MRPL39	39S ribosomal protein L39, mitochondrial		+		7.56	?
16	PABP4	Poly(A)-binding protein 4		+		9.31	[2]
9	PABP3	Poly(A)-binding protein 3		+		9.68	[2]
12	NCL	Nucleolin		+	+	4.60	[45]
3	ANP32A	Acidic leucine-rich nuclear phosphoprotein 32 family member A (putative HLA-DR-associated protein I)		+		3.98	?
3	DHX9	ATP-dependent RNA helicase A (DEAH box protein 9)		+		6.41	[46]
4	HNRNPCL1	Heterogeneous nuclear ribonucleoprotein C-like 1		+		4.93	?
3	HNRNPU	Heterogeneous nuclear ribonucleoprotein U (Scaffold-attachment factor A)		+	+	5.76	?
2	HNRNPA3	Heterogeneous nuclear ribonucleoprotein A3		+		9.10	[47]
4	SYNCRIP	Heterogeneous nuclear ribonucleoprotein Q (Synaptotagmin binding cytoplasmic RNA interacting protein)			+	8.68	?
3	HNRNPA1	Heterogeneous nuclear ribonucleoprotein A1			+	9.17	[48]
2	HNRNPR	Heterogeneous nuclear ribonucleoprotein R			+	8.23	[49]
2	HNRNPA2B1	Heterogeneous nuclear ribonucleoprotein A2/B1			+	8.97	[50]
3	C1QBP	Complement component 1 Q subcomponent-binding protein, mitochondrial (Hyaluronan-binding protein) (Mitochondrial)			+	4.74	[51]
2	SUB1	Activated RNA polymerase II transcriptional coactivator p15 (PC4, RPO2TC1)			+	9.60	?
12	PRPF8	Pre-mRNA-processing-splicing factor 8 (U5 snRNP-specific protein 220 kDa)			+	8.95	[2]
2	EFTUD2	116 kDa U5 small nuclear ribonucleoprotein component			+	4.84	[52]
11	SF3B3	Splicing factor 3B subunit 3			+	5.13	[2, 53]
3	SFRS7	Serine/arginine-rich splicing factor 7			+	11.83	[2, 54]
3	VIM	Vimentin (Cytoskeleton)			+	5.05	[55]
4	IQGAP1	Ras GTPase-activating-like protein IQGAP1 (Membrane)			+	6.08	[56]
		Vesicle/ER/Mitochondrion					
51	CLTC	Clathrin heavy chain 1			+	5.57	[2]
2	CLTCL1	Clathrin heavy chain 2			+	4.29	?
15	HSPA8	Heat shock cognate 71 kDa protein (LPS-associated protein 1, HSP7C) (ER)		+		5.37	[57]
34	HSPA5	Endoplasmic reticulum chaperone BiP (Grp-78, Immunoglobulin heavy chain-binding protein)			+	5.07	[58]
25	HSPA9	Stress-70 protein, mitochondrial (GRP-75, Mortalin)			+	5.87	[2, 57]
9	HSP90AA1	Heat shock protein HSP 90-alpha (Hsp86, NY-REN-38, LPS-associated protein 2) (Membrane)			+	4.94	[59]
7	HSP90AB1	Heat shock protein HSP 90-beta (Hsp84, HSP90B, HSPC2) (Membrane)			+	4.96	[60]
5	HSP90B1	Endoplasmin (GRP-94, Tumor rejection antigen 1)			+	4.76	[61]
4	HSP90AA2P	Heat shock protein HSP 90-alpha A2 (HSP90AA2, HSPCAL3)			+	4.57	[62]
19	CALR	Calreticulin (Calregulin, ER resident protein 60)			+	6.19	[63]
12	P4HB	Protein disulfide-isomerase (cellular thyroid hormone-binding protein)			+	4.76	[64]
6	PDIA6	Protein disulfide-isomerase A6 (ER protein 5)			+	4.95	?
6	PDIA4	Protein disulfide-isomerase A4			+	4.96	?
10	VCP	Transitional endoplasmic reticulum ATPase (Valosin-containing protein)			+	5.14	[65]
5	CALM1	Calmodulin-1			+	4.09	[66]
3	LRPPRC	Leucine-rich PPR motif-containing protein, mitochondrial (GP130)			+	5.81	[67]
3	CANX	Calnexin (Major MHC class I antigen-binding protein p88)			+	4.46	[68]
2	ERO1A	ERO1-like protein alpha (ER oxidoreductase alpha, disulfide bond formation in ER)			+	5.48	?
2	AHSG	Alpha-2-HS-glycoprotein (FETUA, fetuin-A)			+	5.43	[2, 69]
2	COPG1	Coatomer subunit gamma-1 (Golgi)			+	5.32	?
2	COPB2	Coatomer subunit beta			+	5.14	[70]
3	CAPN2	Calpain-2 catalytic subunit (Calcium-activated neutral proteinase 2) (Membrane)			+	4.87	?
2	PRKCSH	Glucosidase 2 subunit beta (Protein kinase C substrate 60.1 kDa protein heavy chain)		+	+	4.33	?
2	PPM1G	Protein phosphatase 1G (PPM1C)			+	4.27	[71]
15	UBA1	Ubiquitin-like modifier-activating enzyme 1 (Mitochondrion)			+	5.49	[2, 72]
2	PSMA7	Proteasome subunit alpha type-7			+	8.60	[73]
2	PSMA2	Proteasome subunit alpha type-2			+	6.91	?
2	PSMA5	Proteasome subunit alpha type-5			+	4.74	?
3	PSMD6	26S proteasome non-ATPase regulatory subunit 6			+	5.45	?
2	PSMD13	26S proteasome non-ATPase subunit 13			+	5.53	[74]
		Cytoskeleton					
21	FLNA	Filamin-A (Actin-binding protein 280)		+	+	5.70	[2, 75]
17	FLNB	Filamin-B (Thyroid autoantigen)		+		5.47	[2]
3	LMNA	Lamin-A/C (Renal carcinoma antigen NY-REN-32)		+	+	6.57	[76]
28	ACTN1	Alpha-actinin-1 (Membrane)			+		[77]
22	ACTN4	Alpha-actinin-4 (Nucleus)			+		[2, 78]
2	ACTA2	Actin, aortic smooth muscle (alpha-actin 2, cell growth-inhibiting gene 46 protein)			+	5.24	?
2	ACTB	Actin, cytoplasmic 1 (Beta-actin) (Nucleus)			+	5.29	[79]
2	ACTBL2	Beta-actin-like protein 2 (Kappa-actin)			+	5.39	?
17	SPTAN1	Spectrin alpha chain non-erythrocytic 1, fodrin alpha chain			+	5.22	[80]
7	MYH9	Myosin-9			+	5.50	[81]
4	TPM4	Tropomyosin alpha-4 chain			+	4.67	[82]
3	TPM1	Tropomyosin alpha-1 chain			+	4.69	[83]
2	TPM3	Tropomyosin alpha-3 chain			+	4.68	[84]
2	TPM2	Tropomyosin beta chain			+	4.66	?
3	BASP1	Brain acid soluble protein 1 (Neuronal axonal membrane protein NAP-22)			+	4.62	?
3	TUBA1C	Tubulin alpha-1C chain (alpha-tublin 6)			+	4.96	[85]
2	TUBB4B	Tubulin beta-2C chain			+	4.79	[86]

^a^Columns left to right: Number of peptides detected by mass spectrometry sequencing (Ab, confirmed by autoantibody staining [2]); Gene name; Protein name; H (high affinity): eluted with 1.0 M salt; M (medium affinity): eluted with 0.6 M salt; L (low affinity): eluted with 0.4 M salt; Predicted isoelectric point (pI); Literature reference reporting autoantibodies against the protein (“?”: no literature evidence found)

### Proteins with medium DS-affinity

From fractions eluted with 0.6 M NaCl from DS-affinity resins, 31 proteins were identified by mass spectrometry (Table 1). Histone H4 and H2B were redundantly identified in both 1.0 M and 0.6 M fractions and thus not counted again. Among these, there are 6 histone autoantigens (H1.0, H1.2, H1.5, H2A.V, H2A.1, and H3.2). Other known protein autoantigens include L5, hnRNP A3, nucleolin, nucleophosmin, lamin A/C, DHX9, PABP4, PABP3, YBX3, DNA-PKcs, PCNA, Ku80 and Ku70, lupus La antigen, filamin A and B, and HSPA8 (Table 1). Several proteins have not been found in literature as reported autoantigens, including SET, PRKCSH, ANP32A, ANP32B, L39, MYBBP1A, and hnRNP protein U and C-like-1.

### Proteins with low DS-affinity

From fractions eluted with 0.4 M NaCl from DS-affinity resins, 69 proteins were identified (Table 1). The 8 proteins that were also identified in the 0.6 M fraction are not counted here. These proteins ranged from nuclear, cytosol, mitochondrial, cytoskeleton, to proteasome. Proteins related to cellular cytoskeleton include tubulin, tropomyosin, actin, alpha-actinin 1 and 4, spectrin, vimentin, calmodulin, calreticulin, and myosin, most of which have been previously reported as autoantigens. Several interesting groups of proteins are identified, including DNA replication 3 licensing factor proteins (MCM6, MCM2, and MCM3), 6 14-3-3 proteins (epsilon, zeta/delta, gamma, theta, beta/alpha, and sigma), 6 heat shock proteins (HSPA5, HSPA9, HSP90AA1, HSP90AA2, HSP90AB1, and HSP90B1), and 5 proteasomal proteins (PSMA2, PSMA5, PSMA7, PSMD6, and PSMD13). Several enzymes that function in the ER were identified, including P4HB, PDIA4, PDIA6, ERO1A, AHSG, and VCP. Other interesting proteins include Golgi proteins (COPG1 and COPB2), mitochondrial LRPPRC, growth factor HDGF, C1QBP, IQGAP1, BASP1, and clathrin.

### DS affinity strongly enriches for autoantigenic proteins

Overall, this study identified a total of 107 proteins from DS-affinity enrichment of HEp-2 cellular extracts (Table 1). Based on current literature, 78 of 107 (72.9%) proteins are confirmed autoantigens. The rest (29 proteins) are potential autoantigens yet to be verified in future studies.

### The DS-affinity HEp-2 proteome shows protein network and functional enrichment

To understand the set of DS-affinity-associated proteins identified in this study, we performed various protein network analyses. From protein association network STRING analysis, 106 nodes (proteins, with HSP90AA2 not recognized) gave rise to 330 edges (protein–protein associations) vs. the expected 142 edges (with average node degree of 6.23, and average local clustering coefficient of 0.531, and PPI enrichment p-value of < 1.0e−16). The STRING analysis results indicate that the set of proteins identified from this study have significantly more interactions among themselves than what would be expected of a random set of proteins of similar size drawn from the genome. This insight suggests that DS-affinity proteins may share certain biological functions. While not definitive, it may also indicate that these proteins share functional or biochemical properties (i.e., forming macromolecular charge affinity complexes) and perhaps immunological properties (we have previously shown that DS-autoAg complexes stimulate autoreactive B cells and autoantibody production [1–4]).

**Fig. 1:**
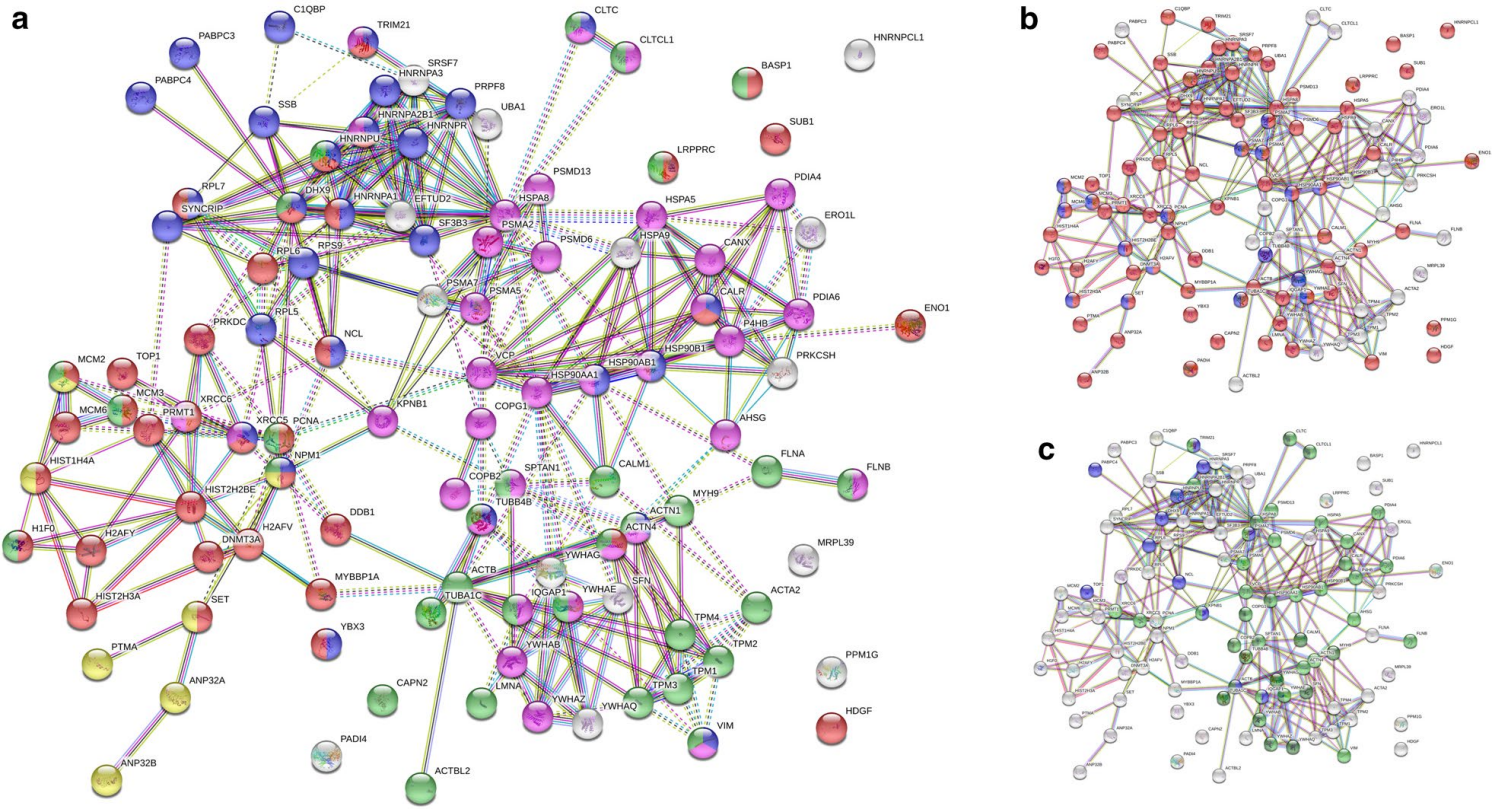
Protein-protein association network of DS-affinity enriched proteins analyzed with STRING. **a** The protein network can be primarily clustered into four clusters: chromosome binding (Red: 36 DNA-binding proteins; Gold: seven histone-binding), 28 RNA-binding proteins (Blue), 35 vesicle components (Purple), and 32 cytoskeleton components (Green). **b** The same network highlighting 82 proteins found in the nucleus (Red, based on GO cellular component) and 17 associated with the mitotic cell cycle (Blue, based on Reactome Pathways). **c** 35 vesicle component proteins (Green) and 20 vesicle-mediated transport proteins (dark Green), and 12 ribonucleoprotein granule (Blue). These classifications are based on GO Molecular Function, GO Cellular Component, and Reactome Pathways. Minimum required interaction score was set to high confidence (0.700) in the network

The proteins identified from this study are not randomly distributed, but rather can be classified into 4 clusters, chromosome-binding, RNA-binding, vesicle, and cytoskeleton (Fig. 1a). Based on GO Molecular Function and Cellular Component analysis, nuclear proteins are the most significant group. Of the 107, 82 proteins can be found in the nucleus, including 36 DNA-binding, 7 histone-binding, and 28 RNA-binding proteins. In particular, 17 proteins are expressed in the M phase of cell cycle (Fig. 1b). According to Reactome Pathways, 24 proteins can be involved in cell cycle, with 20 potentially attributable to the mitotic phase and 17 protein attributable to the G2/M check points. In addition to nuclear proteins, another prominent group is related to vesicles/granulates. This group comprises of 48 proteins, including 35 vesicle components, 20 vesicle-mediated transporters, and 12 ribonucleoprotein granule proteins (Fig. 1c). Another prominent group are proteins associated with cytoskeleton organization (32/107).

**Fig. 2:**
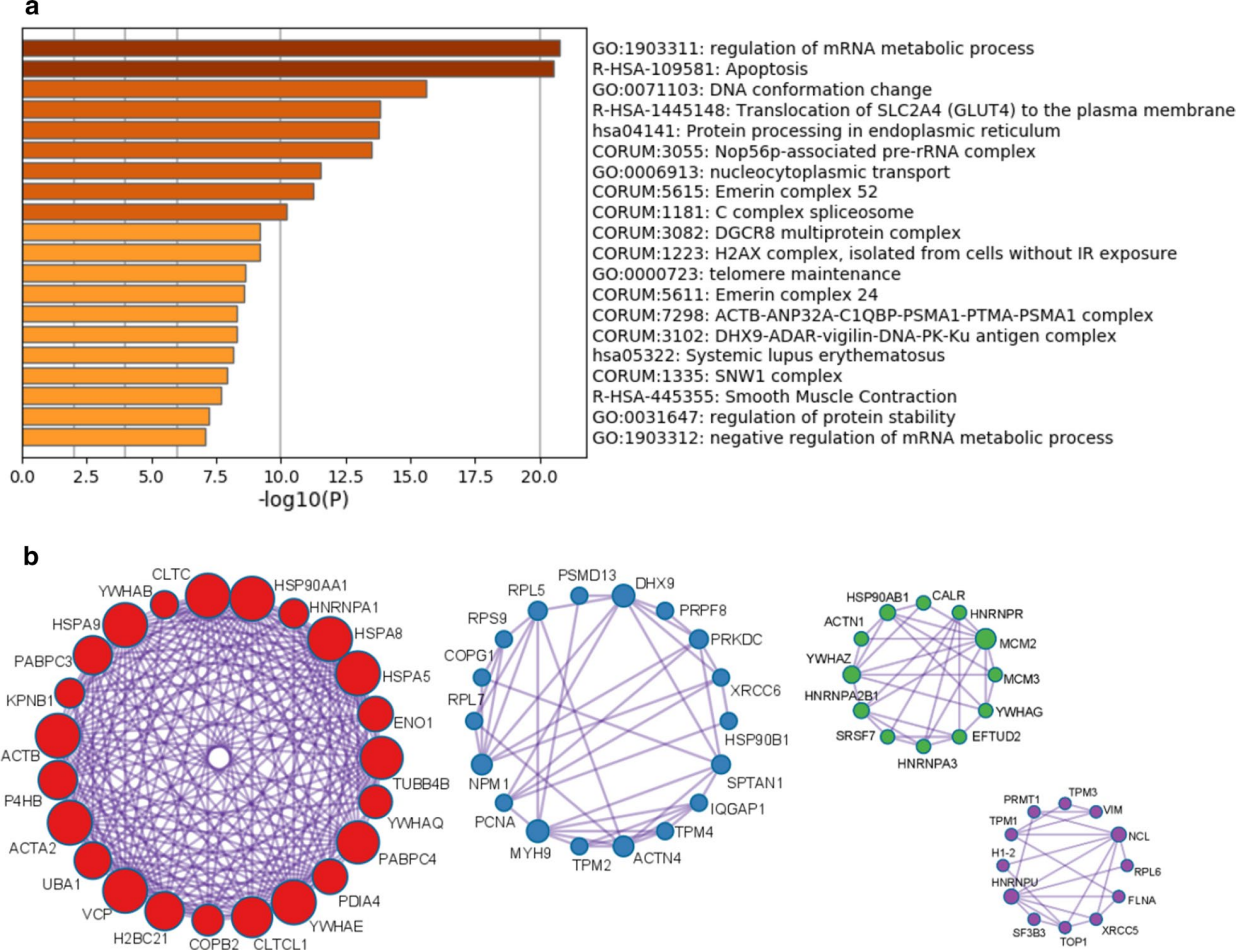
**a** Heatmap of top 20 enriched pathways and processes identified by Metascape. **b** The top four protein–protein interaction networks and components identified by MCODE algorithm of Metascape analysis. Red network is most likely involved in cellular response to heat stress, vesicle-mediated transport, and kinase maturation complex 1. Blue network is most likely involved in PID BARD1 pathway, DHX9-ADAR-vigillin-DNA-PK-Ku antigen complex, and Nop56p-associated pre-rRNA complex. Green network is mostly likely involved in mRNA splicing and C complex spliceosome. Purple network is most likely involved in Nop56p-associated pre-rRNA complex, actin-mediated contraction, and actin filament-based movement

To further capture the relationships among these proteins, Metascape pathway and process enrichment analyses were conducted. The three most prominent ontology clusters identified are mRNA metabolic process regulation, apoptosis, and DNA conformation change (Fig. 2). Other significant clusters include translocation of SLC2A4 to plasma membrane, protein processing in the ER, Nop56p-associated pre-rRNA complex, nucleocytoplasmic transport, Emerin complex 52, C complex spliceosome, DGCR8 multiprotein complex, H2AX complex, telomere maintenance, ACTB-ANP32A-C1QBP-PSMA1-PTMA-PSMA1 complex, DHX9-ADAR-vigilin-DNA-PK-Ku antigen complex, and systemic lupus erythematosus (Fig. 2).

The MCODE (molecular complex detection) algorithm identified four significant networks (Fig. 2). The first MCODE network consists of 23 proteins (CLTC, YWHAB, HSPA9, PABPC3, KPNB1, ACTB, P4HB, ACTA2, UBA1, VCP, H2BC21, COPB2, CLTCL1, YWHAE, PDIA4, PABPC4, YWHAQ, TUBB4B, ENO1, HSPA5, HSPA8, HNRNPA1, and HSP90AA1). This network is likely associated with cellular response to heat stress, vesicle-mediated transport, or kinase maturation complex 1. The second MCODE network consists of 18 proteins (PCNA, MYH9, TPM2, ACTN4, TPM4, IQGAP1, SPTAN1, HSP90B1, XRCC6, PRKDC, PRPF8, DHX9, PSMD13, RPL5, RPS9, COPG1, RPL7, and NPM1). This network is likely associated with PID BARD1 pathway, DHX9-ADAR-vigilin-DNA-PK-Ku antigen complex, or Nop56p-associated pre-rRNA complex. The third MCODE network consists of 12 proteins (CALR, HSP90AB1, ACTN1, YWHAZ, HNRNPA2B1, SRSF7, HNRNPA3, EFTUD2, YWHAG, MCM3, MCM2, and HNRNPR), which are likely associated with mRNA splicing or C complex spliceosome. The fourth MCODE network also consists of 12 proteins (VIM, TPM3, PRMT1, TPM1, H1-2, HNRNPU, SF3B3, TOP1, XRCC5, FLNA, RPL6, and NCL), which are likely associated with Nop56p-associated pre-rRNA complex, actin-mediated cell contraction, or actin filament-based movement.

## Discussion

Autoantigens are the key molecules in autoimmunity and autoimmune diseases, as they are the tissue-resident targets of autoimmune responses and autoimmune diseases. The mechanism by which the normally non-antigenic self-molecules become auto-antigenic holds a key to the understanding of autoimmunity. We proposed that dermatan sulfate has particular affinity for autoAgs and that DS can convert self-molecules to autoAgs by forming DS-autoAg molecular complexes to induce immune responses, and hence any self-molecule with affinity to DS would have the potential to be an autoAg [1]. Using this unifying mechanism of autoantigenicity, we have developed a DS-affinity enrichment strategy to identify potential autoAgs in cell lines and animal organs [2–4]. In this study, we tested whether this strategy would enable us to identify a profile of known autoantigens and perhaps to uncover unknown potential autoantigens from HEp-2 cells.

HEp-2 cells are the classic substrate in clinical tests of autoantibodies for autoimmune diseases [12]. In the indirect immunofluorescence antibody test, microscope glass slides are coated with HEp-2 cells, and human serum is incubated with the HEp-2 cells to allow serum autoantibodies to react with autoantigens in HEp-2 cells. The binding of autoantibodies is detected by fluorescently tagged anti-human Igs and viewed under a microscope. HEp-2 cells give rise to ~ 30 nuclear and cytoplasmic staining patterns associated with various autoimmune conditions (HEp-2 Image Library of University of Birmingham and [12]).

Nuclear patterns from ANAs (antinuclear antibodies) are the most commonly found, ranging from homogeneous, peripheral and nuclear rim, centromere, nuclear pores, to speckled pattern. A few unique cell cycle specific patterns are only identifiable in dividing and mitotic cells, revealing autoantigens such as those expressed in metaphase centrioles and mitotic spindles. Cytoplasmic staining patterns range from Golgi apparatus, mitochondrial, rods and rings, to uncharacterized patterns. Cytoplasmic fiber staining patterns are typically found in association with actin, vimentin, tubulin, cytokeratin, and tropomyosin, all of which we have found in this study.

Despite its wide utility in clinical autoantibody screening for autoimmune diseases, HEp-2 indirect fluorescence test is significantly limited by the lack of molecular specificity. While some patterns are known to be associated with certain autoantibodies/autoantigens, it remains to be better characterized which and how many autoantigens are involved with each pattern. In clinical diagnosis, a particular staining pattern can appear from patients with different autoimmune diseases. For example, a nuclear homogeneous pattern can be from patients with systemic lupus erythematosus, chronic autoimmune hepatitis, or juvenile idiopathic arthritis, and a fine speckled pattern can be from patients with Sjogren’s syndrome, subcutaneous or neonatal lupus erythematosus, or congenital heart block, or other overlap syndrome [12]. For further differentiated diagnoses, samples displaying homogenous patterns are further analyzed by molecular assays such as ELISA for anti-dsDNA, anti-histone, and anti-ENA, whereas samples displaying speckled patterns are further analyzed for anti-ENA, anti-SSA, anti-SSB, and anti-dsDNA.

In this study, we identified various commonly found autoantigens with known associations to different HEp-2 cell staining patterns. For example, histones are mostly involved in homogeneous patterns, and lamin A, B, and C are involved in membranous nuclear rim patterns. Large, variable sized speckles in sponge-like patterns are usually associated with hnRNP, whereas fine speckles are mostly due to SSA/Ro, SSB/La, RNA polymerases and others. Cytoplasmic fiber staining patterns are in association with actin, vimentin, tubulin, cytokeratin, and tropomyosin.

In this study, we also identified a number of interesting potential autoantigens. For example, we identified the family of 14-3-3 proteins that have been reported as autoantigens elsewhere (Table 1), but their association with any particular HEp-2 staining pattern has not been described. As another example, mitotic cell cycle-related patterns have thus far remain poorly understood, whereas our study identified 14 proteins could potentially contribute to mitotic cell staining patterns (PCNA, XRCC5, XRCC6, PRKDC, MCM6, MCM3, MCM2, TUBB4B, IQGAP1, and others) (Fig. 1b). As a third example, COPA mutations impair ER-Golgi transport and cause hereditary autoimmune mediated lung disease and arthritis, and four deleterious variants in the COPA gene (encoding coatomer subunit alpha) were identified in families with an apparent Mendelian syndrome of autoimmunity characterized by high-titer autoantibodies [15]. Circulating anti-COPE (coatomer protein complex subunit epsilon) has been identified as a potential marker for cardiovascular and cerebrovascular events in patients with obstructive sleep apnea [16]. Our study identified two members, COPG1 and COPB2, as potential autoantigens (Table 1).

In addition to cellular staining location, this study also revealed possible functional association of autoAgs identified from DS-affinity. As derived from protein–protein interaction network and pathway analyses, the collection of proteins identified in this study are most likely involved in mRNA metabolism and apoptosis (Fig. 2a). Although the former has no clear role in autoimmunity, apoptosis has established connections to autoimmunity and autoimmune diseases. Apoptosis is well recognized as a source of autoAgs [17]. Our previous study also showed clear evidence that DS is particularly attracted to apoptotic cells and their autoAgs expressed in these cells [1]. In regard to the significant role of apoptosis in autoimmunity, our current findings have reached a consistent conclusion with previous reports by others and us.

It is estimated that there are ~ 20,000 protein-coding genes in the human genome, and ~ 10,000 of these protein-coding genes are expressed in a typical cell. After DS-affinity fractionation, we identified only a small but specific subset 107 proteins (~ 1% of the expressed cellular proteome) from fractions with low to high DS affinity. With 73% of these DS-affinity proteins having verified autoantigenicity, this study demonstrates the powerful feasibility of DS-affinity enrichment for identifying potential autoantigens.

## Conclusions

By DS-affinity enrichment of autoantigens from HEp-2 cellular protein extracts, this study identified 107 proteins, with 78 being verified and 29 being potential autoantigens. These proteins are not a random pool, but rather are clustered in the nucleus, vesicles, and cytoskeleton. This set of proteins shows significantly more interactions than random sets of proteins, revealing apoptosis as a prominent underlying process. Results from this study are consistent with our prior work on autoimmunity [1–5] and provide further support for a more general principle of autoantigenicity on how self-molecules become non-self autoAgs, which may help unravel the molecular etiology of autoimmunity and deepen our understanding of autoimmune diseases. The exact association of these proteins with HEp-2 staining patterns and associated diseases merits extensive investigation in future studies. This study also provides many interesting potential autoantigens for future studies.

## Data Availability

All data generated and analyzed during this study are included in this published article.

## Abbreviations

AutoAg: Autoantigen
DS: Dermatan sulfate
